# An Absorbed-Dose/Dose-Rate Dependence for the Alanine-EPR Dosimetry System and Its Implications in High-Dose Ionizing Radiation Metrology

**DOI:** 10.6028/jres.113.007

**Published:** 2008-04-01

**Authors:** M. F. Desrosiers, J. M. Puhl, S. L. Cooper

**Affiliations:** National Institute of Standards and Technology, Gaithersburg, MD 20899

**Keywords:** alanine, dosimeter, dosimetry, electron paramagnetic resonance, gamma ray, ionizing radiation

## Abstract

NIST developed the alanine dosimetry system in the early 1990s to replace radiochromic dye film dosimeters. Later in the decade the alanine system was firmly established as a transfer service for high-dose radiation dosimetry and an integral part of the internal calibration scheme supporting these services. Over the course of the last decade, routine monitoring of the system revealed a small but significant observation that, after examination, led to the characterization of a previously unknown absorbed-dose-dependent, dose-rate effect for the alanine system. Though the potential impact of this effect is anticipated to be extremely limited for NIST’s customer-based transfer dosimetry service, much greater implications may be realized for international measurement comparisons between National Measurement Institutes.

## 1. Introduction

Alanine-based ionizing radiation dosimetry is firmly woven into the fabric of high-dose radiation metrology. Because of its superior attributes, alanine dosimetry was recognized at an early stage to be of great importance to National Measurement Institute (NMI) services and programs. After many years of use at the NMI level, confidence in the system has grown such that alanine dosimetry use is growing in industry. The transfer of this technology has enabled industrial users of radiation to gain greater control over radiation applications for food/materials processing and medical-device sterilization. When used as a routine dosimeter, alanine dosimetry has proven to be an accurate and robust system, ideal for the often harsh conditions of industrial radiation processing. It is relatively insensitive to a variety of common environmental influences such as humidity, ambient light and temperature. This improves system reliability and reduces laborious/costly troubleshooting events related to other systems commonly used (e.g., radiochromic dosimetry).

From the NMI’s perspective, the broad absorbed-dose range of the alanine system and reported [1] energy and dose-rate independence offered great versatility for calibrating a wide variety of customer-based radiation sources. The National Institute of Standards and Technology (NIST) began developing its alanine system in the early 1990s to replace radiochromic-dye film dosimeters. This work led to the formal establishment of the alanine dosimetry calibration service approximately ten years ago. Over the course of the last decade, routine monitoring of the system revealed a small but significant observation that, after examination, led to the characterization of a previously unknown absorbed-dose-dependent, dose-rate effect for the alanine system. Though the potential impact of this effect is anticipated to be limited for NIST’s customer-based transfer dosimetry service, much greater implications may be realized for international measurement comparisons between NMI’s.

## 2. Calibration of NIST Gamma Sources

### 2.1 Gamma Sources for High-Dose Calibration Services

For industrial high-dose dosimetry calibration services, the measurement quantity of interest is absorbed dose (to water, primarily), reported in units of gray. At NIST, absorbed dose is realized by a water calorimeter in a gamma-ray field produced by a Vertical Beam ^60^Co source [2] with an activity (as of April, 2007) of 48 TBq (1.3 kCi). The technical specifications for this source were described previously [3]. Because the dose rate for this source is relatively low, a high-dose-rate source is needed to perform customer calibrations. The bulk of the services are provided through three Gammacell 220 ^60^Co irradiators (Nordion, Canada).^1^ Their activities are: 37 TBq (1.0 kCi, serial number GC45); 137 TBq (3.7 kCi, serial number GC232); and 666 TBq (18 kCi, serial number GC207), all as of April 2007. Though also available for service operation, the Pool ^60^Co source (0.15 kCi as of April 2007) is rarely requested for calibration work by customers because its low absorbed-dose rate is no longer relevant to industrial needs; however, the Pool source continues to play an important role in the NIST source calibration scheme described here (See 2.3).

### 2.2 NIST Alanine Dosimetry

The alanine dosimetry system has offered great benefits and flexibility to industrial dosimetry and the supporting calibration services. Alanine has a dose range that spans most industrial applications [4]. The alanine system is energy independent (above 100 keV) and, prior to this work, there have been no reports of dose-rate effects. Radiation quality issues are not significant for electrons and photons; recent claims of an electron-photon dose discrepancy [5] lie within the uncertainty of the high-dose certification service. Irradiation temperature and post-irradiation temporal effects have been extensively studied and are minimal [6–8]. Relative humidity effects on the alanine-Electron Paramagnetic Resonance (EPR) measurement [9] are compensated for through the use of an internal EPR reference material [10]. These attributes, together with a rugged commercially manufactured dosimeter and a high-accuracy/precision EPR spectrometer system, enable NIST to operate a postal-based transfer dosimetry system with an expanded uncertainty of less than 2 % (coverage factor, *k* = 2).

The alanine pellet dosimeters currently used in the NIST calibration services are manufactured by Gamma Services (Germany) and distributed through Far West Technology (Goleta, CA). These dosimeters have been used by NIST since the inception of the alanine-based services. The current transfer dosimetry service protocol for the alanine absorbed dose measurement is found in Section 4 of Procedure 12 in the Ionizing Radiation Division (IRD) Quality Manual [11]. For the internal NIST source dose-rate calibrations, the absorbed dose is not computed. As described in the protocol [11], the ratio of the alanine EPR signal amplitude to the ruby reference EPR signal amplitude is normalized to dosimeter mass and averaged for two measurement angles; these dosimeter values, referred to as the dosimeter response, are used to calibrate the dose rate for a fixed irradiation geometry for each radiation source (see Sec. 2.3).

### 2.3 Gamma Source Calibrations

Calibration of the gamma sources within the NIST high-dose calibration facility are performed by measuring the ratio of the alanine dosimeter response for the source being calibrated to that of a reference source. The absorbed dose for these internal calibrations is 1 kGy or less.^2^ This approach simplifies the source comparison to a measurement of two quantities, dosimeter response and time. Absorbed dose is not computed for this calibration exercise because these added steps will introduce additional uncertainties inherent in the calibration curve, and it avoids any issues that might arise from non-linearity in the dosimetry system dose response. Moreover, the very fact that this response-per- time calibration scheme was able to reveal the subtle rate-dependence described here is strong support for the validity of the method.

The four ^60^Co sources described in Sec. 2.1 are used for NIST high-dose calibration services. Prior to 2004, the Pool source and the Gammacells were each calibrated by a direct dosimeter response ratio to the Vertical Beam ^60^Co source [3]. However, the Vertical Beam ^60^Co source dose rate has decayed to a level that requires excessive periods of time (>24 h) to perform comparisons at the absorbed doses (≥1 kGy) routinely used. Since the Vertical Beam ^60^Co source irradiations are performed under water, with the water surface in the vessel exposed to the room environment, there were concerns that a variation in the water level would contribute significantly to the uncertainty of the measurement, as it would be difficult to keep the water level constant for a prolonged period. To address the increasingly longer Vertical Beam irradiation times, modifications to the calibration scheme were developed. In 2004 the calibration scheme was modified so that the Vertical Beam ^60^Co source would be compared only to the Pool source. To improve several aspects of the measurement, the absorbed dose for this comparison was adjusted lower (140 Gy) for the Pool/Vertical-Beam source comparison. The three Gammacells are calibrated by comparison to the Pool source. The Pool source serves well as an intermediary source in the calibration scheme as its dose rate is closer to that of the Gammacells; this permits longer exposure times, resulting in reduced timer uncertainties.

The calibration scheme begins with the known dose rate of the Vertical Beam ^60^Co source, established in 1990 [2]. To transfer that dose rate, eight alanine pellets are irradiated in the calorimeter water tank. The pellets are stacked in a watertight polystyrene cylinder whose axis is fixed perpendicular to the Vertical Beam ^60^Co source at a scale distance of 58.8 cm. The water surface is set at a scale distance of 53.8 cm. This design differs from the published scheme [3]. In the published scheme, this irradiation was done in a polystyrene phantom and a scaling theorem was used to correct for differences in photon interaction cross sections between polystyrene and water. This direct underwater measurement was an improvement as it eliminated scaling theorem uncertainties. In the current calibration scheme the dosimeters are irradiated at the appropriate distance underwater, and no additional corrections are applied to the measured data.

Concurrent to the Vertical Beam irradiation, alanine dosimeters are irradiated to the same absorbed dose (described in [13]) in the absolute center of the isodose region of the Pool-source gamma field. This comparison may be repeated as necessary to achieve an acceptable precision of 0.5 %. The dosimeters are measured using EPR, and the dosimeter response is divided by the irradiation time to convert to units of response•s^−1^. Once the measurements are converted to these common units, the established dose rate in the Vertical Beam source [2] can be used to determine the dose rate in the Pool source.

Similarly, a series of comparisons are made between the dose rates at the center positions of the Pool source and the three Gammacells (GC45, GC232, and GC207) with the alanine transfer vial placed on a polystyrene pedestal set to position the dosimeters in the absolute center of the isodose region of the gamma field. For these comparisons a higher dose is used (e.g., 1 kGy) to reduce the contribution of uncertainty in the timer settings for the highest dose-rate Gammacell. In the GC232 and GC207, irradiations are performed on a pedestal either in a stainless-steel dewar or without a dewar; the dewar is used to improve temperature control at the extremes of the irradiation temperature ranges. The dosimeters are measured and the response•s^−1^ is determined. The center-position dose rate for each Gammacell is determined by comparison to the Pool source center-position dose rate.

It should be noted that the equivalent transit time, the time subtracted from the timer setting that accounts for the absorbed dose received by the dosimeters during the delivery of the dosimeters to and from the irradiation position, is determined for each source. To measure the equivalent transit time, alanine dosimeters are irradiated for a series of very short times. Typically, these times are 5 s, 10 s, 20 s, 30 s, 40 s, and 50 s. The dosimeter response is measured and plotted versus irradiation time. A linear regression of these data is extrapolated to the x axis. The absolute value of the x intercept is the equivalent transit time.

Customer-supplied dosimeters for calibration are routinely irradiated in one of three calibration geometries: ampoule, Perspex, and film block. The rates for each of these positions in the Gammacells (though not all positions are used in each Gammacell), with and without a dewar present, are determined by comparison of the response•s^−1^ for dosimeters irradiated in these positions to the response•s^−1^ for dosimeters irradiated in the center position of the respective Gammacell. This final portion of the calibration scheme remains unchanged from that previously published [3]. As a final check of the dose rates, all irradiation geometries are compared to confirm an equivalent measurement response for dosimeters irradiated to 1 kGy.

### 2.4 Calibration Service Maintenance

#### 2.4.1 Calibration-Source Dose Rates

Approximately annually, dosimeter-response comparisons between the Gammacells, Pool and Vertical Beam ^60^Co sources are performed. The ratios of source dose-rates are determined and ongoing control charts are maintained. These measurements have a standard uncertainty of approximately 0.5 % (*k* = 1).

When possible, dosimetry comparisons are performed between NIST and the high-dose calibration facility of the National Physical Laboratory of the United Kingdom. Dosimeters from each facility are exchanged, measured, and the results compared. Participation in other NMI or multi-NMI international comparisons occurs as appropriate [13].

#### 2.4.2 Transfer Service Dosimetry System Calibration

The alanine dosimetry system is calibrated approximately annually or whenever the EPR measurement configuration changes and/or a new dosimeter lot is used. The details of the calibration procedure are described in the IRD Quality Manual [11].

The calibration curve for the dosimetry system is maintained through the use of check standards. The check standards are alanine dosimeters that have been irradiated to each of the following doses: 1 kGy, 10 kGy, and 50 kGy. These check standards are routinely measured within 48 hours after irradiation, as well as prior to transfer dosimetry service measurements. After the initial calibration of the dosimetry system, these dosimeters are measured periodically (on the average, monthly). Data from these check standards are compiled into a control chart for tracking and comparison. Check standards for doses outside of the 1 kGy–50 kGy range are generated and measured as needed.

#### 2.4.3 Calibration Curve History

In August 2004 the alanine dosimetry system was calibrated using the GC232 gamma source. Shortly thereafter the GC207 gamma source and all its irradiation geometries were calibrated (see Sec. 2.3). These calibrations confirmed the equivalence of response for dosimeters irradiated to 1 kGy in all three NIST Gammacells (GC207, GC232, GC45). For the remainder of 2004 through March 2005, check standards were created using the GC207 because its high dose rate reduced the irradiation time necessary for this work.

## 3. The Alanine Dosimeter Dose/Dose-Rate Effect

### 3.1 Observation of a Dose-Rate Effect

As described in Sec. 2.4, check standards are used to monitor the performance of the dosimetry system used for transfer dosimetry. The measurements are performed to confirm the proper operation of the spectrometer and the reproducibility of the calibration curve. Deviations from the expected values can result from abnormalities in a dosimeter or calibration errors made with the reference radiation source.

A check-standard measurement deviation was noted during a scheduled measurement session in April 2005. After months of check-standard measurements that produced consistent results, a distinct change in the measurement trend was observed on April 1, 2005. This change coincided with a change in the calibration source used to create the check standard. Because check standards are used at relatively high doses, the highest rate source (GC207) was routinely used. On this date, however, the GC232 was used to create check standards because the GC207 was occupied with scheduled irradiations. Because the dose rates in all source geometries are calibrated and found to produce equivalent dosimeter responses at 1 kGy, the sources were considered interchangeable.

**Fig. 1:**
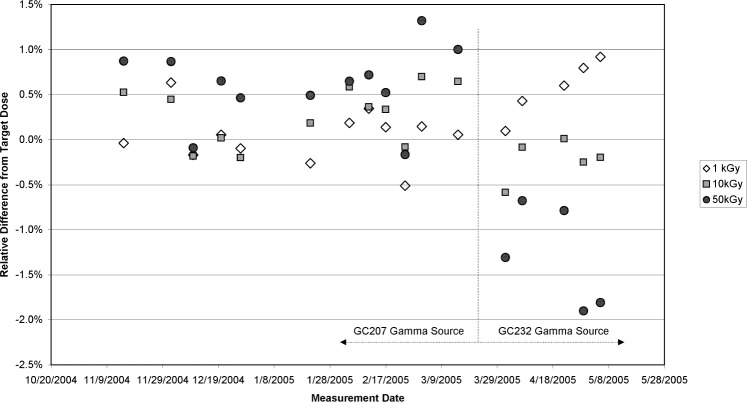
A quality-control check-standard plot for the high-dose transfer dosimetry service. The relative difference of the computed absorbed dose for the check-standard measurement from the absorbed dose delivered is plotted versus the measurement date. Three doses were measured on each date: 1 kGy, 10 kGy, and 50 kGy (the symbols for each are defined in the legend). Dosimeters that measured ± 1 % from the target dose are deemed acceptable.

Figure 1 shows a record of the check-standard measurements from late 2004 through April 2005. The relative difference between the absorbed dose determined from the calibration curve and the dosimeter’s absorbed dose is plotted against the date of measurement. In April 2005, a dose-dependent inversion of the data trend is observed. The 50 kGy measurements that were consistently reading higher than the other doses were now the lowest, and although the 10 kGy doses were lower as well, the decrease was not as dramatic. Curiously, the 1 kGy doses were consistent with the previously measured doses in the chart. This observation initiated a study of the cause for the change in measurement results. Over the course of the month of April the irradiation conditions and records were checked, and the check-standard measurements were repeated several times using the GC232 as the irradiation source (Fig. 1). Concurrent with these measurements, several investigations were conducted to search for a source of error in the irradiation process. Timers (redundant timers are used on each Gammacell) were checked and found to be in working order. The mechanics of the GC232 sample-chamber movement were examined to confirm that the position of the chamber was reproducible at the start of the irradiation, did not move during the course of the irradiation, and did not move from the irradiation position prematurely. Checks of the irradiation geometry included an extensive examination of the dependence of the measurements on the position of dosimeters placed within the sample chamber. No timer or mechanical errors were detected, and the sample-irradiation geometry was confirmed to be correct. The temperature-control measurement and monitoring system for the GC232 and GC207 were also checked and found to be in proper working order.

**Fig. 2:**
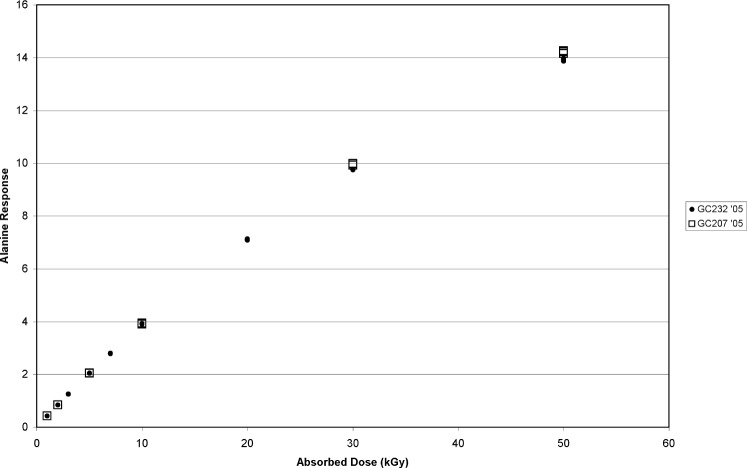
Calibration curve comparison between alanine dosimeters calibrated in the GC207 source (5.052 Gy•s^−1^) and the GC232 source (1.001 Gy•s^−1^) in 2005.

**Fig. 3:**
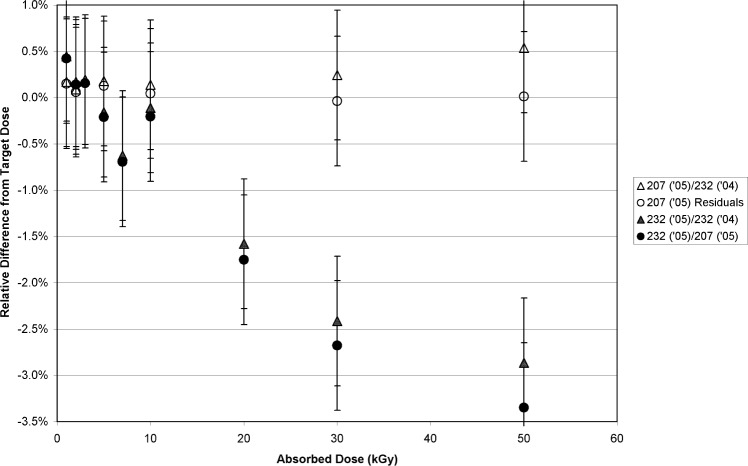
The relative difference of the computed absorbed dose from the absorbed dose delivered is plotted versus the absorbed dose delivered. The dose range is from 1 kGy to 50 kGy. The source of the absorbed-dose measurements used to compute the relative difference is detailed below. The open triangles are 2005 GC207-irradiated dosimeter absorbed doses computed from the 2004 calibration curve created with the GC232 source. The open circles are 2005 GC207-irradiated dosimeter absorbed doses computed from the 2005 calibration curve created with the GC207 source, and represent the residuals of that curve. The filled triangles are 2005 GC232-irradiated dosimeter absorbed doses computed from the 2004 calibration curve created with the GC232 source. The filled circles are 2005 GC232-irradiated dosimeter absorbed doses computed from the 2005 calibration curve created with the GC207 source. The error bars represent the standard uncertainty (*k* = 1).

As a next step in the root-cause analysis, a calibration curve was created in the GC232 (1 kGy, 2 kGy, 3 kGy, 5 kGy, 7 kGy, 10 kGy, 20 kGy, 30 kGy, 50 kGy) and the GC207 (1 kGy, 2 kGy, 5 kGy, 10 kGy, 30 kGy, 50 kGy). Four dosimeters were irradiated at each dose. All sets of calibrated dosimeters were measured by EPR, and a least-squares fit was applied to the data. The function used had the same mathematical form for each curve, which was also the same as the curve established previously in August 2004. A comparison of these two calibration curves is shown in Fig. 2. The differences between the two calibrations are best measured by the relative difference between the measured absorbed doses and (as computed by the source dose rate) the doses delivered (Fig. 3). The open circles represent the 2005 GC207 calibrated dosimeter doses computed from the 2005 GC207 calibration function; these represent the relative residuals (in percent) of the 2005 GC207 calibration curve and are shown for the purpose of comparison. The open triangles in Fig. 3 represent the 2005 GC207 calibrated dosimeter doses computed from the 2004 GC232 calibration function. A large dose-dependent trend is observed in the filled circles that represent the 2005 GC232 calibrated dosimeter doses computed from the 2005 GC207 calibration function. At doses below 10 kGy the GC232 dosimeter doses are mostly consistent with the GC207 dosimeter doses computed from the same (2005) calibration function. However, a significant deviation of the GC232 dosimeter doses from the GC207 dosimeter doses is evident above 20 kGy, and this discrepancy increases as the dose increases to 30 kGy and 50 kGy. The data represented by filled triangles in Fig. 3 represent the GC232 dosimeter doses computed from the 2004 GC232 calibration curve; these data follow the trend of the GC232 dosimeter dose measurements from the 2005 GC207 calibration curve.

**Fig. 4:**
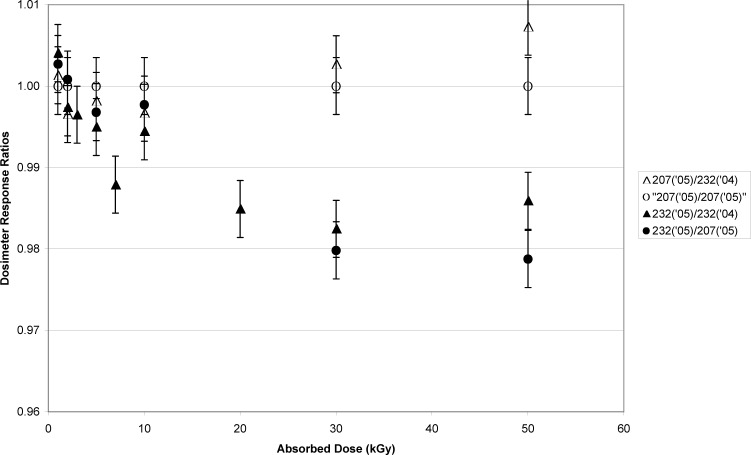
The alanine dosimeter measured response plotted as a ratio to the calibration curve response. The dose range is from 1 kGy to 50 kGy. The source of the ratio measurements is detailed below. The open triangles are 2005 GC207-irradiated dosimeter measurements plotted as a ratio to the 2004 calibration curve created with the GC232 source. The open circles are 2005 GC207-irradiated dosimeter measurements plotted as a ratio to the 2005 calibration curve created with the GC207 source, and are displayed for consistency with Fig. 2. The filled triangles are 2005 GC232-irradiated dosimeter measurements plotted as a ratio to the 2004 calibration curve created with the GC232 source. The filled circles are 2005 GC232-irradiated dosimeter measurements plotted as a ratio to the 2005 calibration curve created with the GC207 source. The error bars represent the standard uncertainty (*k* = 1).

The dosimeter responses were compared directly to verify that the differences between the dosimeter sets were not an aberration caused by the curvature of the dosimeter response in the high-dose saturation range. In Fig. 4 the ratio of the GC232/GC207 dosimeter responses are plotted versus absorbed dose. Clearly, the effect observed in Fig. 3 remains apparent, though the magnitude of the effect is reduced. The difference between these data sets can be attributed to the dose- interpolation step and the saturation of the response in this dose range.

**Fig. 5:**
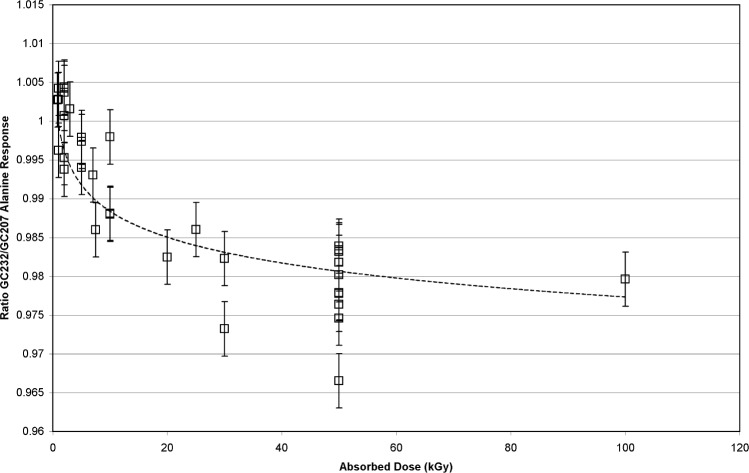
GC232-irradiated alanine dosimeter measurements computed as a ratio to GC207-irradiated dosimeter measurements for the same absorbed dose. The trend line is inserted as a visual aid and is not intended to represent any specific mathematical relation. The error bars represent the standard uncertainty (*k* = 1).

No rate effect is detectable at 1 kGy, and the largest effect is measured above 20 kGy. However, the data at 10 kGy for Fig. 3 and Fig. 4 were not consistent with the measurement trend for doses above and below 10 kGy. A series of additional GC232/GC207 comparisons were measured to better characterize the transition between equivalence and non-equivalence. Figure 5 is a compilation of the Fig. 4 data and additional measurements. From these data, the effect is seen to be significant above 5 kGy.

The 2005 results show that the alanine dosimeters irradiated in the lower-dose-rate (1.001 Gy•s^−1^) GC232 give a response equivalent to alanine dosimeters irradiated in the higher-dose-rate (5.052 Gy•s^−1^) GC207 if the absorbed dose is under 5 kGy. For doses above 5 kGy, the responses of the GC232 and GC207 dosimeters are not equivalent, and the difference in the responses increases with absorbed dose, reaching a maximum above 20 kGy. Of particular interest is the equivalence of dosimeters irradiated (>5 kGy) in the GC232 in 2004 and GC207 in 2005. The dose rate for the GC232 in August 2004 was 1.093 Gy•s^−1^, while the dose rate for the GC207 in April 2005 was 5.052 Gy•s^−1^. However, after the GC232 rate decayed to 1.001 Gy•s^−1^ in 2005, the response of GC232-irradiated dosimeters was ≈2 % (for dosimeters >20 kGy) lower than that of dosimeters irradiated in the GC207 at 5.052 Gy•s^−1^. The comparison of GC232 dosimeters from April 2005 (1.001 Gy•s^−1^) and August 2004 (1.093 Gy•s^−1^) follow this trend of non-equivalence. However, the GC207-irradiated dosimeters in April 2005 (5.052 Gy•s^−1^) are equivalent to the GC232-irradiated dosimeters from August 2004 (1.093 Gy•s^−1^). This relation of the equivalence between the response (**R**) of (>5 kGy) irradiated dosimeters can be summarized as:

$$\begin{array}{l} R(\text{GC}232/’05;\;1.00\;\text{Gy}•s^{-1})\neq R(\text{GC}232/’04;\;1.09\\ \text{Gy}•s^{-1})=R(\text{GC}207/’05;5.05\text{Gy}•s^{-1})=R(\text{GC}207/’04;\\ 5.51\text{Gy}•s^{-1}) \end{array}$$

Regulla and Deffner had claimed alanine dosimetry to be independent of absorbed dose rate [14]. In that work, alanine dosimeters irradiated to 5 kGy, 30 kGy, 100 kGy, and 350 kGy were irradiated with gamma sources of dose rates between 0.028 Gy•s^−^¹ and 28 Gy•s^−1^. Because of the low resolution (a small figure with logarithmic scales) of the graphically displayed data in that publication [14], it remains unclear if the work presented here contradicts or is consistent with the work of Regulla and Deffner. It is not possible to reconcile these findings because the present work did not cover the full range of dose rates previously studied, and the standard uncertainty (0.5 %; *k* = 1) of the measurements presented here is far lower than the standard deviation (≈ 5 %) indicated in the plot of the previous data [14]. However, the measurement data between 0.28 Gy•s^−1^ and 2.8 Gy•s^−1^ in the author’s plot [14] are comparable to the present work and display a trend consistent with the present work. The data of Regulla and Deffner appear to show a lower alanine response for dosimeters irradiated at 0.28 Gy•s^−1^ relative to those at 2.8 Gy•s^−1^. To be conclusive, the dose rate range of the present study would need to be expanded beyond current capabilities.

The data presented here suggest that, for absorbed doses higher than 5 kGy, a rate dependence for alanine is measurable. Moreover, Figs. 3, 4, and 5 demonstrate that the sensitivity of alanine to dose rate (at these doses) becomes significant somewhere between 1.1 Gy•s^−1^ and 1.0 Gy•s^−1^. Though confirmed by a significant number of measurements (Fig. 5), this assertion had to be tested rigorously. Was the effect:

- due to an undetectable mechanical error in the GC232 and GC207?
- unique to the form/composition of the alanine dosimeters used?
- unique to the NIST dosimetry system?

To address these questions, a series of tests using additional gamma sources, other forms of alanine dosimeters, and other dosimetry systems were undertaken.

### 3.2 NIST Gamma-Source Comparisons

The dose/dose-rate effect measurements triggered recollections of previous inconsistencies in source comparisons in 1995 and 2002. In the early days of using alanine dosimetry for internal comparisons at NIST, there was a reliance on expert opinions and publications for certain aspects of the system. One of these aspects was dose rate. Archival publications by recognized experts in alanine dosimetry had found no dependence of the alanine dosimeter on the absorbed-dose rate [14]. Consequently, discrepancies that arose in irradiations of alanine dosimeters at different rates were attributed to other potential sources of error (e.g., environmental influences).

One such discrepancy was noted in 1995. As mentioned in Sec. 2.3, the dose rates for gamma sources used in the NIST calibration scheme were characterized by ratios of the alanine dosimeter responses at doses of 1 kGy and below. However, during an internal calibration in 1995, after multiple measurements at 1 kGy proved reproducible, a check of source comparisons at 25 kGy proved inconsistent with the 1 kGy data (see Table 1). This discrepancy was attributed to unknown environmental influences that presumably influenced the 25 kGy measurements, but not the 1 kGy measurements. Since expert consensus was that dose rate was not an influence on alanine dosimetry, the issue was not pursued further at that time.

The dose-dependent dose-rate effect was again observed in 2002 during another internal calibration (Table 2). Similar results were obtained. There was no observable rate dependence in the alanine response at 1 kGy, but once again at 25 kGy the dosimeters irradiated in the low-rate source under-responded compared to those irradiated in the highest-rate source (GC207).

Resolving these discrepancies was given a low priority because of the significant amount of reproducible 1 kGy comparison data obtained over the course of several years. The differences at 25 kGy were attributed to minor environmental effects related to low-rate long-duration irradiations to high doses. It was thought then that because low-rate sources would not be selected for high-dose irradiations that there would be no impact, or at least an impact that was recognizable and avoidable. What contributed to the recognition of this dose/dose-rate effect was the installation of a quality system at NIST in 2004. Prior to 2004, calibrations of the alanine dosimetry system were more frequent; thus the probability of the effect being observed was low. The frequent pre-2004 recalibration events were accompanied by EPR maintenance routines (e.g., removal/cleaning of EPR sample holders) that prevented long-term comparison measurements. In 2004 NIST began extending the utility of the system calibration to longer times that allowed NIST to directly compare measurements made over months (in this case eight).

**Fig. 6:**
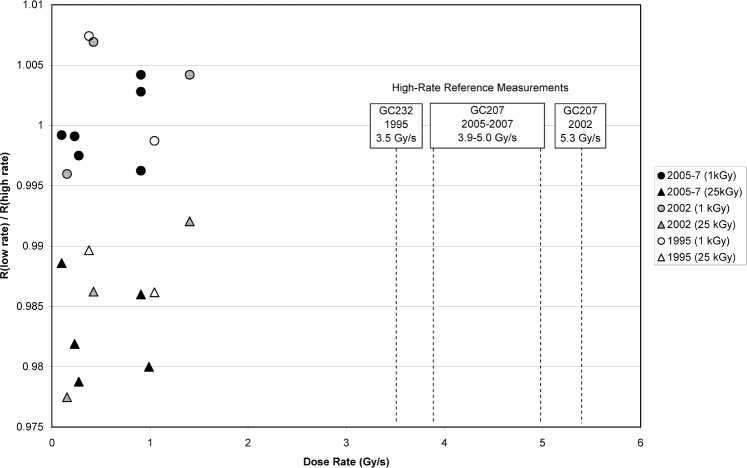
Alanine dosimeter measurement ratios comparing low-dose-rate irradiations to high-dose-rate irradiations at the same dose (1 kGy: circles or 25 kGy: triangles). The measurements made in 1995, 2002, and 2005 are defined in the legend. Inserted into the plot are the dose rates of the high-rate sources in these respective years.

Following the 2005 recognition of the dose/dose-rate effect, several additional comparisons of NIST low-rate sources with the GC207 source were conducted during 2005 through 2007. One example of the data from this period is shown in Table 3; these data correlate well with the previous findings in Tables 1 and 2. The 2005-through-2007 source-comparison measurements are included in Fig. 6 with the results of the 1995 and 2002 data. Figure 6 plots the response ratios of dosimeters irradiated with gamma sources of dose rates lower than 1.4 Gy•s^−1^ (these include the Pool, GC45, and GC232) to dosimeters irradiated concurrently^4^ with gamma sources of dose rates greater than 3.5 Gy•s^−1^. The absorbed doses for these measurements were grouped as low dose (1 kGy) or high dose (predominately 25 kGy, but one 20 kGy and one 30 kGy ratio is included). The 1 kGy ratios of low-rate measurements to high-rate measurements remain clustered about unity consistently from 1995 through 2007 regardless of the gamma source used. The 20 kGy to 30 kGy measurement ratios consistently indicate a response 1 % to 2 % lower than that from the 1 kGy measurements regardless of the gamma source used. These data clearly demonstrate the reproducibility of the dose-dependent dose-rate effect; for more than a decade from 1995 to 2007, dosimeters irradiated to doses higher than 20 kGy with gamma sources of 1.4 Gy•s^−1^ or less gave a lower response than dosimeters irradiated with the same gamma sources to 1 kGy.

### 3.3 Other Alanine Dosimeters

**Fig. 7:**
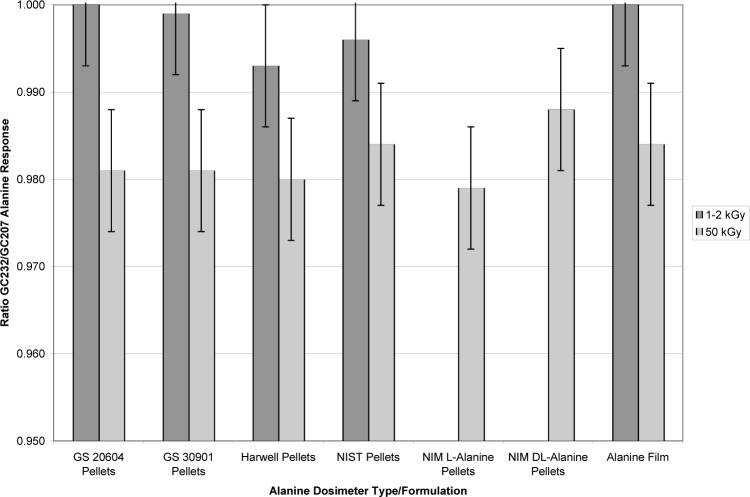
Alanine dosimeter response ratios for GC232/GC207 source comparisons for a variety of alanine dosimeter types and formulations. The dose rates for the GC207 and GC232 sources were 4.58 Gy•s^−1^ and 0.907 Gy•s^−1^, respectively.

The alanine dosimeter comparisons discussed in Sec. 3.2 were conducted with alanine pellets from a single manufacturer (Gamma Service, Germany). Therefore it was important to examine whether the dose/dose-rate effect was unique to this manufacturer, to pellets in general, or to any other alanine dosimeter type. Figure 7 summarizes the study of several alanine dosimeters irradiated to high and low doses in the GC232 and GC207. The dose rates for the GC207 and GC232 were 4.58 Gy•s^−1^ and 0.907 Gy•s^−1^, respectively. The alanine dosimeters were from different manufacturers: Gamma Service (Germany); Harwell (UK); NIST (USA); Bruker (USA); and, NIM (National Institute of Metrology, China).^5^ The study included alanine film and several pellet types including two different lots of Gamma Service pellets and NIM pellets made with either of two alanine stereoisomer forms, L-alanine or DL-alanine (D, L mixture). The alanine dosimeter response ratio measured from dosimeters irradiated in the low-rate GC232 source compared to the high-rate GC207 source were consistently near unity for low-dose irradiations and significantly less than unity for high-dose irradiations. This study demonstrates that the dose/dose-rate effect is inherent to alanine itself regardless of the form or formulation of the dosimeter.

### 3.4 International Comparisons and Tests

The last comparison of the high-dose standards for absorbed dose to water from ^60^Co gamma radiation among the primary dosimetry laboratories maintaining standards and services was performed in the late 1990s. Organized by the Bureau International des Poids et Mesures (BIPM, France) and conducted by the National Institute of Standards and Technology (NIST, USA) and the National Physical Laboratory (NPL, UK), the comparison also included the Istituto Nazionale di Metrologia delle Radiazioni Ionizzanti (ENEA-INMRI, Italy), the Physikalisch-Technische Bundesanstalt (PTB, Germany), the National Institute of Metrology (NIM, China), and the International Atomic Energy Agency (Vienna). Because it does not offer a high-dose service, the BIPM took part at a single dose level (1 kGy) to provide a direct link to the international reference for absorbed dose to water in ^60^Co beams. The comparison was recently published [13] and found that there was a general level of agreement among the institutes at three dose levels: 5 kGy, 15 kGy, and 30 kGy. The agreement was well within the expanded uncertainty for each institute. However, an examination of the level of agreement reveals suggestions of dose-dependent trends in the 5 kGy through 30 kGy data. Also noteworthy is the observation that the best agreement between NIST and NPL occurs at 1 kGy. It is possible that this unknown effect impacted the calibration of high-dose sources maintained by national laboratories. Unfortunately, further analysis of this aspect of the data is not possible because the dose rates for ^60^Co sources within a laboratory’s calibration scheme and the protocol for calibration of these sources are unpublished. It is hoped that future comparisons will include these details so that this effect can be studied in detail.

**Fig. 8:**
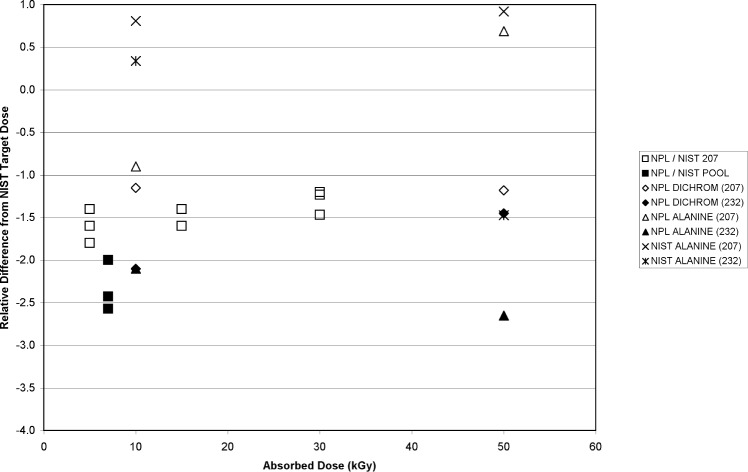
The relative difference of the NPL-computed absorbed dose from the absorbed dose delivered by NIST is plotted versus the absorbed dose delivered. The open squares are 5 kGy, 15 kGy, and 30 kGy doses delivered by the GC207 source. The filled squares represent a 7 kGy dose delivered by the Pool source. The open diamonds are NPL dichromate doses irradiated in the GC207 source to 10 kGy or 50 kGy. The filled diamonds are NPL dichromate doses irradiated in the GC232 source to 10 kGy or 50 kGy. The open triangles are NPL alanine doses irradiated in the GC207 source to 10 kGy or 50 kGy. The filled triangles are NPL alanine doses irradiated in the GC232 source to 10 kGy or 50 kGy. The × symbols are NIST alanine doses irradiated in the GC207 source to 10 kGy or 50 kGy. The * symbols are NIST alanine doses irradiated in the GC232 source to 10 kGy or 50 kGy. The standard uncertainties (*k* = 1) for these measurements are: NPL alanine, 1.2 %; NPL dichromate, 1.04 %; NIST alanine, 1.0 %.

Periodic comparisons with other NMI’s is an important component of the NIST IRD Quality System. Historically, NIST has performed high-dose comparisons with NPL. The goal is to perform these comparisons at least biannually. For these comparisons, NIST and NPL exchange 12 alanine dosimeter vials, irradiate four vials to each of three doses (5 kGy, 15 kGy, 30 kGy), and return the vials to the issuing laboratory for analysis. Typically, there is a 1 % to 2 % relative difference between the absorbed dose as measured by the analyzing laboratory and the absorbed dose delivered by the irradiating laboratory. The results from the most recent comparison in December 2004 are displayed in Fig. 8. Because prior to this comparison NIST had recently changed its calibration scheme to employ the Pool source for routine internal comparisons, NIST made a small modification to the protocol. Instead of irradiating four dosimeter vials to 5 kGy, 15 kGy, and 30 kGy in its highest rate source (GC207, 5.052 Gy•s^−1^), NIST irradiated three vials to each of the three prescribed doses and irradiated the remaining three vials to 7 kGy in the lower rate Pool source (0.110 Gy•s^−1^). Figure 8 shows that the 5 kGy, 15 kGy, and 30 kGy GC207-irradiated dosimeters, as measured by NPL, are relatively consistent with regard to the relative difference (approximately 1.5 %) between the NPL-assessed dose and the NIST-delivered dose. However, the Pool-irradiated dosimeters deviated from this trend and yielded a relative difference of approximately 2.3 %. The net 0.8 % relative difference is consistent with the rate effect measured at NIST for 7 kGy (Fig. 5).

The final step in the dose/dose-rate study is independent verification of the measurements made at NIST. To verify the effect, NPL kindly agreed to supply and measure alanine and dichromate dosimeters to be irradiated by NIST. Using the NIST five-position hold er, the experimental design for each run was to irradiate two NPL dichromate dosimeter ampoules and two NPL alanine dosimeter vials, with a NIST alanine dosimeter vial in the fifth position.^6^ Two sources were used for the irradiation, the GC207 (4.578 Gy•s^−1^) and the GC232 (0.907 Gy•s^−1^). The doses selected for each source were 10 kGy and 50 kGy (10 kGy was the lowest dose possible dose for this dichromate dosimeter formulation).

**Fig. 9:**
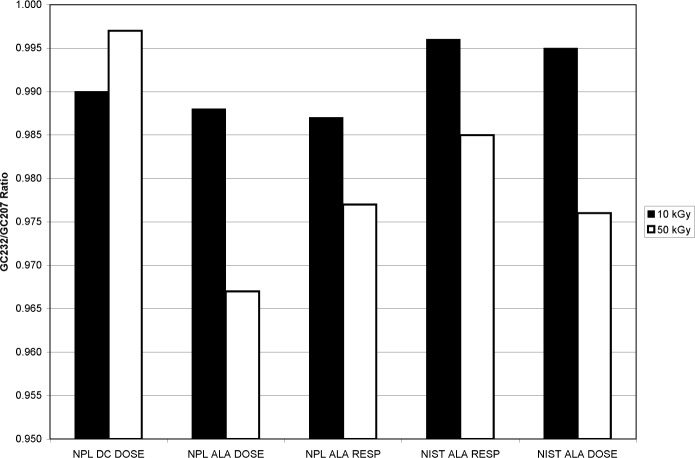
Dosimeter ratios for GC232/GC207 source comparisons for: NPL dichromate doses, NPL alanine doses, NPL alanine EPR amplitudes, NIST alanine EPR amplitudes, and NIST alanine doses. The black bars are 10 kGy ratios and the white bars are 50 kGy ratios. The standard uncertainties (*k* = 1) for these measurements are: NPL alanine, 1.2 %; NPL dichromate, 1.04 %; NIST alanine, 1.0 %.

The ratio of the mean NPL-measured dose (9.79 kGy; uncertainty of 1.2 %, *k* = 1) for the two alanine vials irradiated to 10 kGy in the GC232 source to the mean NPL-measured dose (9.91 kGy) for the two alanine vials irradiated to 10 kGy in the GC207 source was 0.988 (Fig. 9). The ratio of the mean NPL-measured dose (9.79 kGy; uncertainty of 1.04 %, *k* = 1) for the two dichromate ampoules irradiated (together with the NPL alanine cited above) in the GC232 source to the mean NPL-measured dose (9.89 kGy) from the two dichromate ampoules irradiated (together with the NPL alanine cited above) in the GC207 source was 0.990 ± 0.008. The alanine dosimeter GC232/GC207 response ratio for NPL dosimeters measured above was 0.987 ± 0.009. The alanine dosimeter response GC232/GC207 ratio for NIST dosimeters irradiated simultaneously with the NPL dosimeters to 10 kGy was 0.996 ± 0.004; the GC232/GC207 ratio for NIST-measured absorbed dose was 0.995 ± 0.007.

The ratio of the mean NPL-measured dose (48.7 kGy) from the two alanine vials irradiated to 50 kGy in the GC232 source to the mean NPL-measured dose (50.3 kGy) from the two alanine vials irradiated to 50 kGy in the GC207 source was 0.967 (Fig. 9). The ratio of the mean NPL-measured dose (49.3 kGy) from the two dichromate ampoules irradiated (together with the NPL alanine cited above) in the GC232 source to the mean NPL-measured dose (49.4 kGy) from the two dichromate ampoules irradiated (together with the NPL alanine cited above) in the GC207 source was 0.997 ± 0.008. The alanine dosimeter GC232/GC207 response ratio for NPL dosimeters measured above was 0.977 ± 0.009. The alanine dosimeter response GC232/GC207 ratio for NIST dosimeters irradiated simultaneously with the NPL dosimeters to 50 kGy was 0.985 ± 0.004; the GC232/GC207 ratio for NIST-measured absorbed dose was 0.976 ± 0.007.

Though slightly higher than the previously determined ratios shown in Fig. 5, the NIST response ratios at 10 kGy (0.996) and 50 kGy (0.985) are consistent with the previous findings in that the 50 kGy ratio is about 1 % lower than the 10 kGy ratio. The NIST alanine, NPL alanine and NPL dichromate dose ratios are consistent with each other at 10 kGy. However, at 50 kGy the NPL dichromate system measured the absorbed dose for dosimeters irradiated in the GC232 and GC207 sources to be equivalent, while NPL alanine dosimeters irradiated simultaneously with the NPL dichromate dosimeters measured an approximately 2 % lower dose in the GC232 source as compared to the GC207 source NPL alanine dosimeter dose measurement. So, whereas the 50 kGy NPL dichromate dosimeter showed no effect of irradiation at different rates, the NPL alanine dosimeter measurements confirmed the rate effect documented by NIST alanine dosimeter measurements and are consistent with all other alanine dosimeter types shown in Fig. 7.

### 3.5 EPR Studies

Elucidation of the physico-chemical mechanism for the dose/dose-rate dependence is beyond the scope of this study; however, a series of EPR investigations were undertaken to learn if spectroscopic data could support the hypothesis that the differences in EPR signal amplitude are related to differences in the free-radical composition of alanine dosimeters irradiated to identical doses but at different dose rates.

Certain aspects of alanine dosimetry suggest different behavior of the chemistry at low doses versus high doses. For example, there is a marked difference in the temporal changes of the EPR signal amplitude after irradiation below and above 1 kGy [8]. Though no significant change in the EPR spectrum was measurable at high doses compared to low doses, low-intensity signal contributions to the overall spectrum from secondary or tertiary radicals could not be ruled out. Early experimental studies using photo-bleaching [16] and spin-trapping [17] of irradiated alanine were able to detect spectroscopically different radicals at high doses. Later, advances in EPR spectral analysis identified a secondary radical and postulated the existence of a third [18]. A dose dependence of the relative proportions of these radicals has not been reported.

Starting with the assumption that one or more of the alanine free radicals is rate sensitive and that rate sen sitivity is dose dependent, experiments were conducted that sought measurable spectroscopic differences in the EPR spectrum. There are several EPR parameters that vary with the molecular composition or configuration of a free radical. The most common of these, g-factor and hyperfine coupling constants, are not well resolved because the irradiated alanine spectrum is composed of two or more radicals with multiple broad resonances. Microwave power saturation behavior is also known to be dependent on the form of the free radical. The EPR signal amplitude is proportional to the square root of the applied microwave power, being approximately linear with power until saturation is achieved at higher power. For different free radicals, the power dependence can exhibit differences in the power value at maximum EPR amplitude and in the general shape of the EPR amplitude versus power curve over the range of applied microwave power. Based on the magnitude of the effect measured in this study, these differences were assumed to be relatively small, so rather than compare absolute differences a comparison of the ratios of the power dependences for high- and low-dose-rate irradiated alanine pellets was measured.

The absorbed doses selected for the study were: 100 Gy, 1 kGy, 5 kGy, 20 kGy, and 50 kGy. At each dose level, a four-dosimeter vial was irradiated simultaneously in both the GC45 source (0.873 Gy•s^−1^) and the GC207 source (4.41 Gy•s^−1^). The irradiation timers were set such that the irradiations ended at approximately the same time. The dosimeters were measured between 24 hours and 48 hours after irradiation. For each of the four dosimeters irradiated to the same dose but with different dose rates, the signal amplitude was computed as a function of microwave power. The microwave power was varied by stepping the attenuator in 1 dB increments to yield 21 power levels from 0.5 mW to 50 mW.^7^

**Fig. 10:**
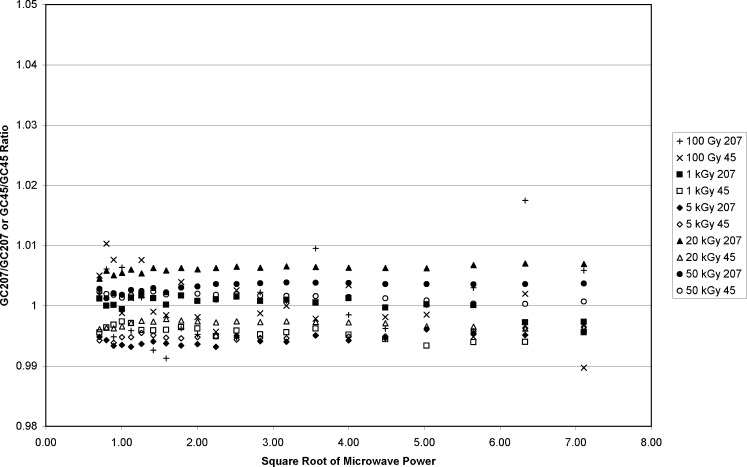
Alanine EPR amplitude measurement ratios for individual dosimeters irradiated in like sources (e.g., GC207 compared to GC207) for doses from 100 Gy to 50 kGy. The EPR amplitudes are plotted against the square root of the incident microwave power of the EPR spectrometer. The GC45/GC45 ratios are the × symbol and open symbols. The GC207/GC207 ratios are the + symbol and filled symbols.

**Fig. 11:**
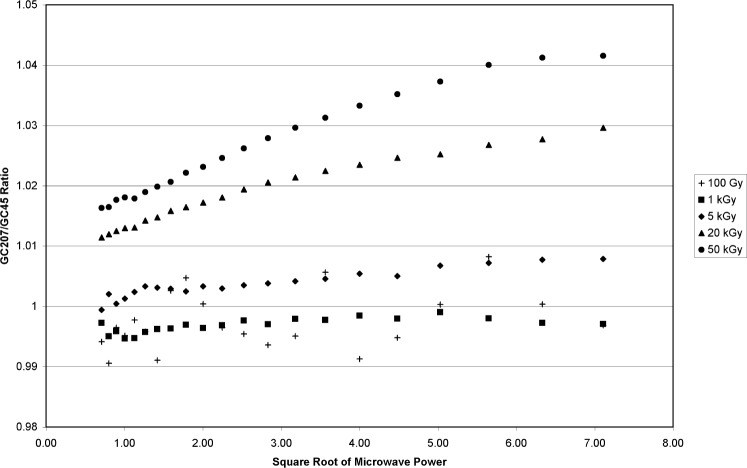
Alanine EPR amplitude measurement ratios for individual dosimeters irradiated in different sources (GC207 compared to GC45) for doses from 100 Gy to 50 kGy. The EPR amplitudes are plotted against the square root of the incident microwave power of the EPR spectrometer. The absorbed doses for the GC207/GC45 ratios are given in the legend.

The microwave power dependence for each dosimeter was recorded and combined as a ratio for dosimeters irradiated with either of the two gamma sources. Figure 10 shows a plot of representative measurements from these combined ratios, but only for dosimeters irradiated with identical rates. GC207 dosimeters were compared to other GC207 dosimeters and the same for GC45 dosimeters. There are no observable trends regardless of the gamma source at each dose level or between dose levels. However, when ratios are computed for dosimeters irradiated to the same dose with different dose rates (Fig. 11) there is a significant divergence in the data that increases with increasing absorbed dose. For the response ratios measured in Fig. 5, the dose/dose-rate effect on alanine becomes significant above 5 kGy. This trend holds true in Fig. 11, with significant differences in the power dependence at 20 kGy and 50 kGy. The 100 Gy and 1 kGy GC207/GC45 ratios in Fig. 11 are consistent with the 100 Gy and 1 kGy data in Fig. 10, where the ratios were for dosimeters of the same dose and dose rate. The GC207/GC45 ratio at 5 kGy, as in Fig. 5, suggests the onset of change. At 20 kGy and 50 kGy the ratios rise above unity because of the reduced EPR amplitude for the GC45 irradiated dosimeters at high dose. In addition, the GC207/GC45 ratio of EPR amplitudes for dosimeters irradiated to 20 kGy and 50 kGy increases with microwave power. This is a result of the differences in the microwave-power saturation curves for the different radical distributions contained in the respective dosimeters. This effect indicates that the free-radical composition of the high- and low-dose-rate irradiated alanine dosimeters is different at doses higher than 5 kGy. This does not necessarily imply different (or new, unique) radicals, but could indicate different relative concentrations of the radicals that are present at lower doses. The EPR signal amplitude of the central resonance in the alanine spectrum is a combination of the EPR intensities from two or more free radical types. The relative sensitivity of each free-radical type to microwave power produces changes in the overall EPR intensity that results from the combination of the individual intensities of each radical type.

It may be possible to exploit the relative response differences that are dependent on microwave power to discern whether measurements of dosimeters irradiated above 5 kGy are influenced by the rate effect. During industrial processing the movement of product accompanied by dosimeters to, from, and about the radiation source exposes the dosimeters to a broad range of dose rates. The extent to which, as part of this process, low dose-rate exposures contribute to the total absorbed dose and influence the dose assessment is unknown. A microwave-power dependence comparison of dosimeters irradiated in an industrial facility to calibrated dosimeters irradiated at a fixed (high) dose rate may be a diagnostic tool to explore rate-effect influences in industrial radiation dosimetry.

## 4. Summary

This work characterizes a complex relation between the radiation chemistry of crystalline alanine and the applied dose rate as a function of absorbed dose. This effect appears intrinsic to alanine itself and is not dependent on the form or formulation of the alanine dosimeter. These studies suggest that the production of one or more of the alanine radicals is dependent on the dose rate and that this rate effect becomes significant above 5 kGy. These data are certainly consistent with other alanine dose dependent effects that have been observed in the kilogray range. Dose-dependent behavior has been documented for effects based on temperature [6], time [8], and relative humidity [9]. The boundary for all of these effects is above 1 kGy and may point to a common origin in the chemistry of the different free radicals that combine to form the measured spectrum.

Typically high-dose-rate calibration sources maintained by NMIs are calibrated against low-dose-rate sources that are used to realize the gray with a primary standard (e.g., calorimeter). If a dose rate is established for a high-rate gamma calibration source by comparison to dosimeters calibrated in a low-rate source at absorbed doses in the kGy range, errors could be introduced in the dose rate for the high-rate calibration source. If this calibration source is in turn used for service work, a dose-dependent error would be further transferred to industrial customers. For the NMI that has ensured that their dose rates are not influenced by this dose-rate effect, two considerations remain. First, caution would be advised to avoid using alanine dosimeters at high doses (> 5 kGy) for transfer calibrations of low-dose-rate (< 2 Gy•s^−1^) sources and second to avoid using a gamma source with a low-dose-rate to calibrate alanine dosimeters.

It is a distinct possibility that high-dose calibration services provided by other NMIs and/or secondary standards laboratories are in error due to the rate effect, and that this error has been propagated to customers. These laboratories should perform internal system checks that use the experimental design employed in the present work, and follow that with comparisons to verify their rates. Considering that the previous international high-dose dosimetry comparison [13] did not take this effect into account, it is recommended that for follow-up NMI comparisons the calibration schemes of the participants should be examined prior to starting.

## Figures and Tables

**Table 1:** Alanine dosimeter response ratios for source comparisons in the 1995 internal calibration. Gamma source identifiers are accompanied by their dose rate in Gy•s^−1^. The values are accompanied by a Type A uncertainty representing the measurement standard deviation.

Absorbed Dose (kGy)	Pool (0.3783)	GC45 (1.045)
	GC232 (3.453)	GC232 (3.453)
1.0	1.007 ± 0.004	0.999 ± 0.004
25	0.990 ± 0.004	0.986 ± 0.004

**Table 2:** Alanine dosimeter response ratios for source comparisons in the 2002 internal calibration. Gamma source identifiers are accompanied by their dose rate in Gy•s^−1^.^3^ The values are accompanied by a Type A uncertainty representing the measurement standard deviation.

Absorbed Dose (kGy)	Pool (0.1544)	GC45 (0.4242)	GC232 (1.404)
	GC207 (5.348)	GC207 (5.348)	GC207 (5.348)
1.0	0.996 ± 0.004	1.007 ± 0.004	1.004 ± 0.004
25	0.977 ± 0.004	0.986 ± 0.004	0.992 ± 0.004

**Table 3:** Alanine dosimeter response ratios for 2006 source comparisons. Gamma source identifiers are accompanied by their dose rate in Gy•s^−1^. The values are accompanied by a Type A uncertainty representing the measurement standard deviation.

Absorbed Dose (kGy)	Pool (0.0994)	GC45 (0.2744)	GC232 (0.907)
	GC207 (4.578)	GC207 (4.578)	GC207 (4.578)
1.0	1.001 ± 0.004	1.003 ± 0.004	0.996 ± 0.004
50	0.985 ± 0.004	0.988 ± 0.004	0.983 ± 0.004

## Footnotes

1 Certain commercial equipment, instruments, or materials are identified in this paper to foster understanding. Such identification does not imply recommendation or endorsement by the National Institute of Standards and Technology, nor does it imply that the materials or equipment identified are necessarily the best available for the purpose.

2 The alanine EPR signal amplitude is linear with absorbed dose up to ≈4 kGy.

3 The similarity of the GC207 dose rates for 2002 and 2004 is not an error. In mid 2004, the source was reloaded with higher activity Co-60 and recalibrated in late 2004.

4 The tandem irradiations were timed such that they would terminate at approximately the same time and permit the post-irradiation readout times to be equivalent.

5 NIST pellets were manufactured according to the published preparation [16]. NIM pellets were manufactured according to the published preparation [7]. The L and DL designation refers to the stereoisomeric forms of alanine pellets used, L-alanine and DL-alanine, respectively. The quantity of available NIM pellets was insufficient to permit low-dose comparisons.

6 An examination of the position dependence with alanine dosimeters for this five-position geometry in the GC232 and GC207 revealed a relative standard deviation of 0.50 % and 0.25 % respectively.

7 The EPR amplitude is a ratio of the alanine EPR amplitude of the center resonance and the ruby reference material EPR amplitude (as described in [10]). Other alanine EPR recording parameters include: frequency, 9.684 GHz; center field, 345.5 mT; magnetic-field sweep width, 1.0 mT; modulation amplitude, 0.2 mT; time constant, 1.3 s; sweep time, 41.9 s.
